# Patient experience and satisfaction after same-day discharge radical prostatectomy using a personalized, digital perioperative programme

**DOI:** 10.1007/s00345-024-05099-7

**Published:** 2024-06-18

**Authors:** Daniele d’Agate, Alberto Martini, Marine Lesourd, Christophe Tollon, Guillaume Loison, Christophe Almeras, Benjamin Pradère, Ambroise Salin, Jean-Baptiste Beauval, Guillaume Ploussard

**Affiliations:** 1 Urology Department, La Croix du Sud Hospital, 52b, Chemin de Ribaute, 31130 Quint Fonsegrives, France; 2 Department of Surgical Sciences-Urology, Città Della Salute E Della Scienza Di Torino, Molinette Hospital, University of Turin, 10126 Turin, Italy

**Keywords:** Prehabilitation, Surgery, Remote monitoring, eHealth

## Abstract

**Purpose:**

To assess the patient experience and satisfaction after the implementation in routine of a personalized, digital programme before and after same-day discharge (SDD) robot-assisted radical prostatectomy (RARP).

**Methods:**

The study is a pre/post-interventional, multi-surgeon, unicentre, prospective study. All consecutive patients undergoing SDD RARP were included during a 6-month period. After a pre-interventional assessment of the satisfaction rate (n = 26), all patients (n = 46) were introduced to the Betty. Care platform and followed the BETTY COACHING programme which included a specific radical prostatectomy module. The primary endpoint was patient satisfaction 6 weeks after SDD RARP. Secondary endpoints were hospital stay, readmission and complications rates, unplanned visits, and remote monitoring data.

**Results:**

Median age and PSA were 66 years and 7.0 ng/ml. Lymph node-dissection and nerve-sparing procedures were performed in 41.3 and 87.0% of patients, respectively. Median operative time and blood loss were 80 min and 150 ml, respectively. The 90-day rates of unplanned visits, readmission and complications were improved after the digital tool implementation (2.2, 2.2, and 8.7%, respectively). Mean satisfaction score was 9.6 out of 10 (8.0 before implementation). Median duration of pain was 2 days after discharge, with median pain intensity of 2/10. Median duration of daily active use of remote monitoring was 34 days. The urinary continence rate was 91.3% 6 weeks after surgery in the postinterventional cohort.

**Conclusions:**

The implementation of a personalized, surgery-specific, digital programme combining prehabilitation, patient education, rehabilitation, patient-reported outcome measurement and remote monitoring, improves patient experience and satisfaction and could help promoting early discharge even after a major surgery.

## Introduction

Several studies have assessed the safety of same-day discharge (SDD) robot-assisted radical prostatectomy (RARP) in routine practice [1–5]. Although different countries’ experience demonstrated comparable rates of complications between inpatient and outpatient surgery, the spread of this hospitalization type remains limited. Nationwide French data and large North American population-based studies have reported a weak SDD penetration, less than 5% in an overall RARP population [3, 6]. This low proportion of SDD cases could be explained by weak patient adherence and/or unoptimized perioperative pathways. Moreover, very few data on patient-reported experience and satisfaction have been reported after SDD RARP [1, 7, 8].

A large amount of data suggests that better patient preparation and care coordination at home could meaningfully improve outcomes and patient experience after surgery and facilitate early discharge [9]. We previously demonstrated that the implementation of a dedicated prehabilitation programme increased the acceptance of SDD RARP in routine practice without compromising postoperative outcomes [10]. However, such on-site prehabilitation programmes could be challenging to launch and maintain because of motivational issues for care teams, the absence of financial and incentive support for such programs, and the lack of human resources in health care systems [11].

Previous studies have highlighted that, although RARP patients were globally satisfied after surgery, efforts should be focused on patient education prior to surgery to facilitate early mobilization and return to work and to improve patients’ experience [12]. Thus, efforts should be made to secure post-discharge follow-up thanks to rehabilitation programmes and remote monitoring. Electronic health (eHealth) has emerged as a promising tool to enhance adherence to educational programs and treatment outcomes [13–15]. Moreover, eHealth can be used throughout the whole perioperative setting without the need for excessive human effort and financial support.

Herein, we prospectively evaluated the impact on patient satisfaction and experience after SDD RARP of a personalized, digital, perioperative programme called BETTY COACHING (https://betty.care), including prehabilitation, rehabilitation, electronic patient-reported outcome/experience measurements (ePRO/PREM), and remote monitoring, and a radical prostatectomy-specific module.

## Materials and methods

The study is a post-interventional, multi-surgeon, unicentre, prospective study, conducted in accordance with Good Clinical Practice and the Declaration of Helsinki. The study was approved by a national Ethics Committee (IRB: IRB00010835/ COS-RGDS-202112001), and waived the requirement for informed consent from patients. All consecutive patients undergoing SDD RARP were included during a 6-month period. Exclusion criteria for SDD RARP have been described previously [16, 17]. Briefly, SDD was not offered to patients under oral anticoagulation or having a distance home-hospital > 150 km. All patients were managed according to an institutionally-approved enhanced recovery after surgery protocol without modifications during the study period, except the implementation of the digital programme. The minimal follow-up for data analysis was 3 months after surgery.

The pre-interventional cohort included consecutive patients before the implementation of the digital programme in order to evaluate the patient satisfaction rate after SDD surgery. The aim was to calculate the number of needed patients during the post-interventional phase. Thus, we prospectively assessed the satisfaction of 26 consecutive SDD patients (median age: 63.7 years) before the implementation of the digital pathway (non-BETTY cohort). The satisfaction was 8.0/10 on a visual analogic scale (standard deviation: 2.7). The sample size was calculated to detect a 1.0/10 increase in satisfaction after the digital pathway implementation (statistical power of 80%; unilateral test; alpha value of 0.05) which meant an objective of 46 patients to be included in the BETTY COACHING cohort. These 46 patients were included in the BETTY COACHING cohort consecutively to the non-BETTY cohort patients.

The patients were introduced to the Betty. Care platform by their surgeon who also assigned the specific radical prostatectomy module, in addition to the common pathway (BETTY COACHING). Details regarding the functions and compliance to this platform have been described here: 10.1089/tmr.2024.0012. The patients were then fully autonomous for downloading the app (France Connect system and free access) and in the use of the platform after an initial connection. The patient app included checklists before key moments, alerts for starting or stopping activities, educational materials (podcasts, videos on physical activities, articles) for improving patient information and condition before and after surgery including surgery-specific physiotherapy exercises. ePRO/PREMs were prospectively collected to record patient satisfaction, experience, and specific outcomes, such as continence and sexual function questionnaires.

The primary endpoint was patient satisfaction 6 weeks after SDD RARP. Satisfaction scale (from 0 to 10) was fulfilled by the patient the day before the post-operative visit, 6 weeks after RARP in both cohorts. Secondary endpoints were hospital stay, readmission, prolonged care at discharge, unplanned visits, and remote monitoring data (including daily reports of pain level after discharge, analgesics consumption, temperature). Readmission was defined by any hospitalization at emergency and/or surgery units. Urinary continence was defined by the use of 0 or 1 safety pad per day. Pain was evaluated in-app with a visual analogic scale (from 0 to 10). Perioperative complications were reported according to the Clavien–Dindo classification [18]. SPSS 22.0 software (SPSS, Inc, Chicago, Illinois) was used for analysis.

## Results

Baseline characteristics are listed in Table 1. Median (IQR) age and BMI were 66 (60–68) years and 25.8 (24.1–28.7) kg/m2. Median (IQR) PSA was 7.0 (5.4–9.1) ng/ml. Lymph node-dissection and nerve-sparing procedures were performed in 41.3% and 87.0% of patients, respectively.

**Table 1:** Baseline characteristics

	n = 46
Age, years Median (IQR)	66.0 (60.2–67.9)
ASA score (%):	
2	41 (89.1)
3	5 (10.9)
Ongoing antiplatelet therapy (%)	8 (17.4)
BMI, kg/m2 Median (IQR)	25.8 (24.1–28.7)
PSA, ng/ml Median (IQR)	7.0 (5.4–9.1)
Prostate volume, cc Median (IQR)	35.0 (26.2–51.2)
Biopsy ISUP (%)	
2	31 (67.4)
3	10 (21.7)
4–5	5 (10.9)
cN1 (%)	1 (2.2)
Operative time, minutes Median (IQR)	80.0 (70–95)
Blood loss, ml Median (IQR)	150.0 (100–250)
Lymph node dissection (%)	19 (41.3)
Nerve-sparing surgery (%)	40 (87.0)
pT stage (%)	
pT2	31 (67.4)
pT3a	13 (28.3)
pT3b	2 (4.3)
pN1 (%)	2 (4.3)
Pathological ISUP (%)	
2	28 (60.8)
3	11 (23.9)
4–5	7 (15.2)

*BMI* body mass index, *ISUP* International Society of Urological Pathology, *PSA* prostate-specific antigen, *ASA* American Society of Anesthesiologists

Median (IQR) operative time and blood loss were 80 (70–95) minutes and 150 (100–250) ml, respectively. Pathological staging revealed pT3, pN1 and ISUP 4–5 disease in 32.6, 2.2, and 15.2% of patients, respectively.

Post-operative outcomes are shown in Table 2. The 90-day rates of unplanned visits, readmission and complications were 2.2, 2.2, and 8.7%, respectively. Mean satisfaction score was 9.6 out of 10 (median 10; IQR 9–10).

**Table 2:** Post-operative outcomes and remote monitoring data in the BETTY cohort. Comparisons of outcomes with the non-BETTY cohort (n = 26)

	n = 26	BETTY cohort n = 46
Primary endpoint		
Patient satisfaction (SD)^τ^	8.0 (2.7)	9.6 (0.5)
Secondary endpoints		
Unplanned visit (%)	3 (11.5)	1 (2.2)
Readmission (%)	1 (3.8)	1 (2.2)
Complications (%)	5 (19.2)	4 (8.7)
Grade 1	1 (3.8)	2 (4.3)
Grade 2	4 (15.4)	2 (4.3)
Remote monitoring data^a^		
Duration of pain (days)	N/A	2.05 (1.6)
Maximal pain level	N/A	2.37 (2.8)
Average pain level	N/A	2.26 (3.5)
Days under oral analgesics		
Full dose	N/A	1.0 (2.0)
Partial dose		0.8 (1.7)
Duration of remonte monitoring (days)	N/A	29.0 (15.2)
Reported temperature > 38.5 °C	N/A	0 (0)

Pain level measured by analogic scale

*SD* standard deviation

^τ^p = 0.037

^a^Reported as means (standard deviation)

In an exploratory analysis, comparisons were made with the previous 26 RARP patients who had not followed the digital pathway. The use of digital pathway led to non-significant benefits in all secondary endpoints.

**Fig. 1:**
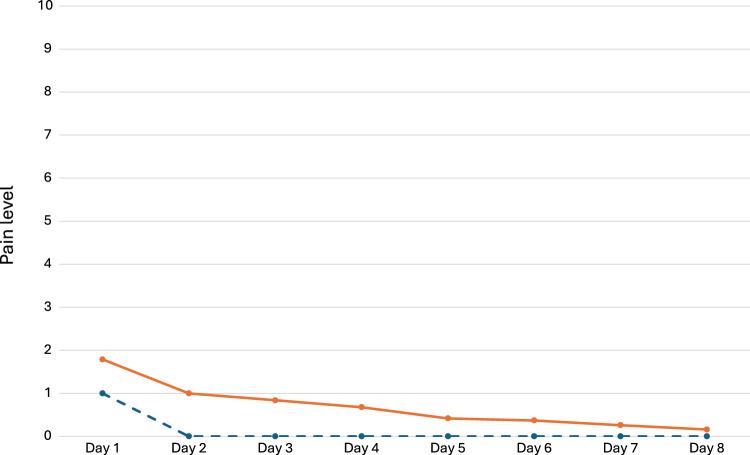
Pain level evolution over time after discharge. Orange line: mean values; grey dotted line: median values. Pain level assessed in-app by a visual analogic scale

Remote monitoring data are also listed in Table 2. No post-operative fever was reported. On average, patients used full dose analgesics during 1 day at home (median 0.5; IQR 0–2), and a partial dose during 0.8 day (median 0; IQR 0–1.75). Median duration of pain was 2 days (IQR 0.75–4.5), with median pain intensity of 2/10 (IQR 0.75–3.25). Evolution of pain level over time after discharge is illustrated in Fig. 1 as median and mean values.

The median (IQR) duration of daily active use of remote monitoring through the app was 34 (10–45) days.

The urinary continence rate was 91.3% (42 patients with 0 or 1 safety pad) 6 weeks after surgery.

## Discussion

Over the past decade, same-day discharge (SDD) has emerged as a reasonable hospitalization regimen after robot-assisted radical prostatectomy [4, 5, 17].

Literature currently depicts different countries and health care system experience, with almost comparable outcomes [2, 5, 6]. Globally, SDD did not lead to worse post-operative results in terms of complication or readmission rates and appeared as safe as inpatient procedure. Some studies even noted a better safety profile, regarding the rate of post-operative infection and functional recovery after RARP [2, 6]. Although current trends show a wider adoption of SDD, penetration rate remains scarce in routine. Nationwide French data demonstrated that less than 2% of annual RARP are performed as a SDD procedure [3]. In a large population-based study using the National Surgical Quality Improvement Program Database, approximately 3.7% of RARP patients underwent outpatient surgery [6].

However, recent data showed that the adoption of SDD can reach higher rates in expert high volume centers [2, 4, 19]. Surgeon and hospital volume are key factors for this generalization, as well as optimized perioperative pathways [10, 11].

To date, few data have been published regarding the patient satisfaction to SDD RARP [1, 8]. A recent longitudinal prospective study including 72 RARP patients reported that SDD did not affect satisfaction compared with patients staying 1 or 2 nights at hospital after surgery [7]. In a larger comparative series of 392 consecutive patients, Cheng et al. reported that median satisfaction scores were similar between outpatient versus inpatient surgery patients [2]. Another study has suggested that most patients expressed low regret about choosing the SDD pathway for RARP [8]. In a study of 72 patients, 65.7% felt no regret about their decision of choosing the SDD RARP protocol and 90.3% of men would have made the same decision. Moreover, 97.1% of patients would also recommend SDD to others. Interestingly, the authors have evaluated the factors associated with regret. Patients experiencing postoperative pain expressed a higher regret. We also previously found a significant correlation between pain and SDD failure in the first published multicentre SDD study [16]. In a large cohort of more than 350 patients, pain was an independent predictor of SDD failure, highlighting the need for adapting analgesia protocols and for closely monitoring pain even after discharge. In the present study, our findings were reassuring with patients using full dose of pain killers during only 1 day on average at home. Mean duration of in-app reported pain was only 2 days. These outcomes suggested that the perioperative programme was in accordance with SDD pathway, allowing a safe day 0 discharge without compromising patient experience.

In-app patient preparation prior to surgery thanks to prehabilitation and education programmes could also help to minimize side effects, improve patients’ postoperative psychological and physiological status, and ease recovery after surgery. Indeed, Betty.care is to our knowledge the first digital tool aiming to integrate various materials and functionalities including prehab- and rehabilitation protocols, demographics and medical history questionnaires, checklists, ePRO collection, and remote monitoring, providing by this way a holistic approach to surgery. We previously assessed the good patient adherence to this digital platform. Patient compliance to all in-app materials was also high, > 75% in all domains, and reaching 100% for the initial questionnaires. Moreover, we did not observe a significant drop in patient compliance over time within the post-operative follow-up. Mean duration of in-app daily monitoring was 29 days in the present series, confirming the good patient compliance to daily remote monitoring.

Satisfaction was collected in-app thanks to a visual analogic scale. We chose this assessment to automate a patient-reported satisfaction measurement, without intervention from the surgical team which can induce interpretation biases. In a recent study, patients have reported that the collection of PROs by a smartphone app was easier or equivalent to the traditional approach reinforcing the interest of remote monitoring for increasing the patient adherence to PRO collection [13].

Moreover, the use of digital platforms could lead to a wider adherence to outpatient surgery and to a fast-track procedure allowing faster discharge, which may result in cost reductions at the hospital- and healthcare levels. Recently, Nijland et al. conducted a prospective study combining same-day discharge with remote monitoring. A success rate of 88 percent was achieved. The authors noted that specific preparation in the preoperative pathway was a positive factor contributing to this success [20]. Our experience was also in line with these considerations and highlighted the importance of a combination of prehabilitation, enhanced recovery after surgery, rehabilitation protocols, as well as a fully organized at-home discharge.

## Conclusions

The implementation of a personalized, radical prostatectomy-specific, digital programme combining prehabilitation, patient education, rehabilitation, patient-reported outcome measurement and remote monitoring, improves patient experience and satisfaction and could help promoting same-day discharge procedure after robot-assisted radical prostatectomy.

## Data Availability

Data available on request.
